# A Novel Three-Step Extraction Strategy for High-Value Products from Red Algae *Porphyridium purpureum*

**DOI:** 10.3390/foods10092164

**Published:** 2021-09-13

**Authors:** Tao Li, Jin Xu, Weinan Wang, Zishuo Chen, Chulin Li, Hualian Wu, Houbo Wu, Wenzhou Xiang

**Affiliations:** 1 CAS Key Laboratory of Tropical Marine Bio-Resources and Ecology, Guangdong Key Laboratory of Marine Materia Medica, Institution of South China Sea Ecology and Environmental Engineering, South China Sea Institute of Oceanology, Chinese Academy of Sciences, Guangzhou 510301, China; taoli@scsio.ac.cn (T.L.); wangweinan0220@163.com (W.W.); 18390943716@163.com (Z.C.); lchlxpy@126.com (C.L.); hlwu@scsio.ac.cn (H.W.); wuhoubo@scsio.ac.cn (H.W.); 2 Southern Marine Science and Engineering Guangdong Laboratory (Guangzhou), Guangzhou 511458, China; 3 CAS Key Laboratory of Renewable Energy, Guangdong Provincial Key Laboratory of New and Renewable Energy Research and Development, Guangzhou Institute of Energy Conversion, Chinese Academy of Sciences, Guangzhou 510640, China; xujin@ms.giec.ac.cn

**Keywords:** *Porphyridium purpureum*, fractional extraction, phycoerythrin, polyunsaturated fatty acid, polysaccharides

## Abstract

The microalga *Porphyridium* accumulates high-value compounds such as phycoerythrin, polyunsaturated fatty acids, and polysaccharides, and thus, the extraction of these compounds could significantly expand the value of *Porphyridium* biomass. In the present study, a novel fractional extraction strategy based on the characteristics of these compounds was established using cold water, 95% ethanol, and hot water. The yield of phycoerythrin, lipids, and polysaccharides was 63.3, 74.3, and 75.2%, respectively. The phycoerythrin exhibited excellent fluorescence characteristics but had low purity. The crude lipid was dark with poor fluidity. Digalactosyldiacylglycerol and sulphoquinovosyldiacylglycerol containing C20:5 and C20:4 were the most abundant glycerolipids, while glucose, xylose, and galactose constituted the intracellular polysaccharides that had covalently bound to proteins (8.01%), uronic acid (4.13%), and sulfate (8.31%). Compared with polysaccharides and crude lipids, crude phycoerythrin showed the best antioxidant activity. Overall, the three-step fractional extraction process was feasible for *Porphyridium*; however, further purification is necessary for downstream applications.

## 1. Introduction

*Porphyridium*, a unicellular microalga that belongs to phylum Rhodophyta, class Bangioideae, order Bangiales, and family Porphyridiaceae, live in seawater, brackish water, freshwater, and moist soil environments [1]. Of the nine documented species of *Porphyridium*, *Porphyridium purpureum* (also called *Porphyridium cruentum*) has been widely studied as a model species. This red alga can accumulate high-value compounds, such as B-phycoerythrin, polyunsaturated fatty acids (PUFAs), and intracellular polysaccharides, the potential feedstock for food, cosmetics, and drugs [2]. After phycoerythrin extraction, the PUFAs and intracellular polysaccharides still remain in the algal residue. However, no extraction method has been reported so far for the simultaneous extraction of these three high-value compounds from *Porphyridium* biomass.

Phycoerythrin, the major photosynthetic pigment in *P. purpureum*, exhibits several biological activities, including anti-oxidation, anti-tumor, and immunity enhancement [1]. In addition, phycoerythrin is widely acknowledged as a rare orange-red pigment in nature due to its strong coloration and antioxidant activity similar to blue phycocyanin from *Spirulina* [1]. Currently, phycocyanin is extensively used in pigments and health care products [3]. The commercial high-purity phycoerythrin, which has been widely used as a fluorescent dye, is primarily derived from large red algae but in a very low content (<0.1% of dry weight, DW). Furthermore, its purification process is complicated and incurs high cost [4]. In contrast, *Porphyridium* possesses high phycoerythrin content (~8% DW) and could be cultivated on a large scale [2,5]. Therefore, extracting phycoerythrin from *Porphyridium* might reduce the cost and promote the market value of phycoerythrin [4]. In 2021, the market price of 1 mg of highly pure (A_545_/A_280_ > 5.0) B-phycoerythrin was USD 741.4 [6]. The common extraction methods for phycoerythrin include microwave-assisted extraction, in-situ stirring extraction, and the freezing/thawing method, which involves −20 °C freezing for 2 h, followed by thawing at room temperature (RT, 20–25 °C) [7]. Owing to the light and pH sensitivity, phycoerythrin extraction requires mild conditions [8]. The fatty acids in the total lipid (9–14% of DW) of *P. purpureum* are composed of PUFAs (40%), such as arachidonic (C20:4, ARA) and eicosapentaenoic (C20:5, EPA) acids, a potential omega-3 source without fish oil [9,10]. The oil derived from *Nannochloropsis* has been approved by the US Food and Drug Administration (FDA) as an EPA sment [11]. Some products are already selling *Nannochloropsis* powder with >3.0% DW EPA content at USD 95.0/Kg [12]. The *Porphyridium* lipids are rich in EPA and ARA, and it is expected to develop more valuable products than *Nannochloropsis*. Ethanol is a cost-effective and safe polar organic solvent for lipid complexes [13]. Previous studies have used ethanol for lipid extraction from oleaginous microalgae [13,14]; however, it was never explored for *Porphyridium* lipids. Intracellular polysaccharides constitute >50% of *P. purpureum* DW [5]. Studies have proved that *Porphyridium* polysaccharides have excellent antioxidant, moisturizing activity and immunity-enhancing and anti-viral activities. The polysaccharides of *Porphyridium* are currently used in cosmetics [1]. Recent studies have reported that polysaccharides can combat COVID-19, but the related drugs are yet to enter the market [15]. Acid, alkaline, and water extraction methods are commonly used for polysaccharide extraction. However, the purity and activity of the final product vary with the extraction method [16]. Moreover, most polysaccharides extraction methods require heating which might destroy the phycoerythrin and PUFAs in *Porphyridium*. Therefore, the PUFAs and phycoerythrin are extracted first before the polysaccharides.

The crude phycoerythrin extract contains a large number of other proteins [7]. Similarly, the ethanol extract has impurity of fat-soluble small molecule compounds (e.g., triacylglycerol, chlorophyll, and carotenoids) [17]. Nevertheless, many industries, such as health care products, cosmetics, and pigments, do not have high purity requirements for the additives as they can be directly utilized in the downstream applications. On the contrary, the antioxidant activity of the additives significantly influenced by the extraction process is one of the important bioactivities [18]. For instance, heating accelerates the lipid extraction rate, but it aggravates the oxidation of microalgae PUFAs [19]. Although the crude extracts of *Porphyridium* can be used directly, the extraction process also demands antioxidant activity preservation, which can be used as a feasibility factor.

In this study, a novel fractional extraction method was established for phycoerythrin, lipid, and intracellular polysaccharide extraction from *Porphyridium* biomass that includes: (1) in-situ stirring extraction of phycoerythrin at low temperature; (2) in-situ stirring extraction of lipid with ethanol at RT; and (3) in-situ stirring extraction of intracellular polysaccharides by heat. Finally, the characteristics and activities of the crude extracts were assessed to explore the feasibility of the above process. This research can facilitate the commercialization of *Porphyridium*.

## 2. Materials and Methods

### 2.1. Microorganisms and Culture Conditions

*P. purpureum* SCS-02 was isolated by our laboratory [5]. It was cultured in an outdoor open pond for 20 days. Biomass was harvested using a Q45 tubular centrifuge (Guangzhou Fuyi Liquid Separation Technology Co., Ltd., Guangzhou, China) and stored at −20 °C after freeze-drying using an FD-1-50 freeze dryer (Boyikang, Beijing, China).

### 2.2. Fractional Extraction Process of Active Substances

#### 2.2.1. Phycoerythrin

Approximately 50 mL of deionized water (1:50, *v*/*v*) was added to 1.0 g of freeze-dried *Porphyridium* powder. The mixture was extracted at 4 °C for 6 h with stirring using a magnetic rotor. The phycoerythrin extract (supernatant) was obtained by removing the solid mass (1st algal residue) through centrifugation at 6000× *g* rpm for 5 min. The 1st algal residue was cleaned with deionized water and freeze-dried as described above.

#### 2.2.2. Crude Lipid

The 1st freeze-dried algal residue was added to 40 mL of 95% ethanol for extraction at RT for 6 h with magnetic rotor stirring. The supernatant was collected by centrifugation at 6000× *g* rpm for 5 min and the ethanol was removed by a RE-2000 rotary evaporator (Yarong Biochemical Instrument Factory, Shanghai, China). The remaining residue, named 2nd algal residue, was cleaned with deionized water and freeze-dried as described above.

#### 2.2.3. Intracellular Polysaccharide

The 2nd freeze-dried algal residue was added to 40 mL of deionized water and the mixture was extracted at 80 °C for 2 h with stirring. Then, the supernatant was collected by centrifugation at 6000× *g* rpm for 5 min, and subsequently precipitated with 100% ethanol overnight. After 12 h, the floating floccules of intracellular polysaccharides were collected. Subsequently, the crude polysaccharides were deproteinized using trichloroacetic acid to obtain semi-refined polysaccharides. As earlier, the remaining algal residue (3rd algal residue) was cleaned with deionized water and freeze-dried as described above.

### 2.3. Phycobiliprotein Content

The phycobiliprotein content in the extract was estimated by a TU-1810 spectrophotometer (Persee Instrument Co., Ltd., Beijing, China) at 620, 550, and 565 nm. The contents of phycocyanin, allophycocyanin, and phycoerythrin were estimated using the following equations [5]:

PC (mg mL^−1^) = (A_620_ − 0.7 × A_650_)/7.38 (1)

APC (mg mL^−1^) = (A_650_ − 0.19 × A_620_)/5.56 (2)

PE (mg mL^−1^) = (A_565_ − 2.8 × PC−1.34 × APC)/12.7 (3)

where PC, APC, and PE are the concentrations of phycocyanin, allophycocyanin, and phycoerythrin, respectively. A_620_, A_650_, and A_565_ are the absorbance of crude phycoerythrin extracts at 620, 650, and 565 nm, respectively.

### 2.4. Lipid Composition

Ten mg of the crude lipid was put into a 2 mL centrifuge tube and mixed with 750 μL chloroform/methanol solution (2:1, *v*/*v*, −20 °C) by vortex for 2 min. The mixture was placed on ice for 40 min, and then, 0.19 mL ddH_2_O was added by vortex for 30 s. This process was followed by centrifugation at 12,000× *g* rpm for 5 min at RT. Subsequently, 300 μL of the lower layer was transferred into a new centrifuge tube. Meanwhile, the second extraction was performed using 500 μL of chloroform/methanol solution and combined with the previous 300 μL. The total 700 μL was vacuum dried and then re-dissolved in 200 μL of isopropanol. The mixture was filtered through a 0.22 µm membrane before liquid chromatography–mass spectrometry (LC–MS) analysis.

The separation of lipid fractions was conducted on a Ultimate 3000 high performance liquid chromatography system (Thermo Fisher, Dreieich, Germany) equipped with an ACQUITY UPLC^®^ BEH C_18_ column (100 × 2.1 mm, 1.7 µm, Waters, Shanghai, China). The mobile phase consisted of solvent A (acetonitrile: water = 60:40; 0.1% formic acid + 10 mM ammonium formate) and solvent B (isopropanol: acetonitrile = 90:10; 0.1% formic acid + 10 mM ammonium formate). An increasing linear gradient of solvent A (*v*/*v*) was used as follows: 0–5 min, 70.0–57.0% A; 5.0–5.1 min, 57.0–50.0% A; 5.1–14.0 min, 50.0–30.0% A; 14.0–14.1 min, 30% A; 14.1–21.0 min, 30.0–1.0% A; 21.0–24.0 min, 1% A; 24.0–24.1 min, 1.0–70.0% A; and 24.1–28.0 min, 70% A. The flow rate was 0.25 mL min^−1^.

The ESI-MSn experiments were performed on the Thermo Q Exactive Focus mass spectrometer with the spray voltage of 3.5 and −2.5 kV in the positive and negative modes, respectively. Sheath gas (nitrogen) and auxiliary gas (nitrogen) were set at 30 and 10 arbitrary units, respectively. The capillary temperature was 325 °C. Full scans (*m*/*z* 150–2000) were performed by the Orbitrap analyzer scanned at a mass resolution of 35,000. The normalized collision energy was 30 eV. High energy collision dissociation (HCD) scans were performed for MS/MS data-dependent acquisition. Dynamic exclusion was implemented to remove noise from the MS/MS spectra.

### 2.5. Biochemical Composition of Biomass

The algal powder was hydrolyzed with 0.5 N NaOH at 80 °C for 20 min, and the process was repeated thrice. Protein content was analyzed colorimetrically using the Lowry method [20]. Ten mg of the algal powder was hydrolyzed with 6 mL of 1.0 N H_2_SO_4_ at 80 °C for 1 h, and the process was repeated thrice. The total carbohydrate content was measured using the phenol-sulfuric acid method [21]. Total lipid content was determined using the modified Khozin-Goldberg method [22]. The freeze-dried algal powder (or residue) was combusted at 550 °C for 10 h, and the ash content was determined as follows:

Crude fiber content (%DW) = 1 − Pro − Cho − Ash − Lipid(4)

where Pro, Cho, Ash, and Lipid are the protein, total carbohydrate, ash, and total lipid content, respectively.

### 2.6. Determination of Monosaccharide Composition

Five mg of the polysaccharides was hydrolyzed with 2.0 mol L^−1^ trifluoroacetic acid at 110 °C for 2 h, followed by repeated co-distillation with methanol until dry. Additionally, hydroxylamine hydrochloride, inositol, and pyridine were added to the hydrolysate at 90 °C for 30 min in an oscillating water bath, followed by the addition of acetic anhydride at 90 °C for 30 min. The mixture was cooled, and the aldononitrile acetate derivatives of saccharides were separated [23]. The monosaccharide components were determined by a GC-2014 gas chromatography system (Shimadzu, Kyoto, Japan) equipped with an SH-Rtx-5 capillary column (30 m × 0.25 mm × 0.25 μm, Shimadzu Kyoto, Japan). Argon was used as the carrier gas and the column temperature was programmed from 120 to 210 °C at 3 °C min^−1^. The temperature of the injection port and detector were 250 and 280 °C, respectively. The injection volume was 1.0 μL with a split ratio of 30:1.

### 2.7. Determination of Uronic Acid and Sulfate Radical Content

Uronic acid content was determined using the meta-hydroxyphenyl method [24]. Sulfate content was determined according to the method described by Zhang et al. (2000) [25]. The polysaccharides were hydrolyzed with 2 mL of 1 M hydrochloric acid at 100 °C for 6 h, and the mixture was filtered through a 0.45 μm microporous membrane. The final volume (5 mL) was adjusted with distilled water before sulfate content estimation by ICS-2500 ion chromatography (Dionex Corp., Sunnyvale, CA, USA).

### 2.8. Determination of Antioxidant Activity

The antioxidant activities of the extracts were measured as free radical scavenging activity for DPPH (2, 2-diphenyl-1-picrylhydrazyl), ABTS (2, 2′-azinobis 3-ethylbenzothiazoline-6-sulfonic acid), and superoxide anion. Ascorbic acid was used as a positive control.

#### 2.8.1. ABTS Radical Scavenging Ability

The crude phycoerythrin was dissolved in distilled water to prepare 0.3, 0.6, 0.9, 1.2, 1.5, and 1.8 mg mL^−1^ solutions. The crude lipid was dissolved in ethanol to prepare 0.5, 1.0, 1.5, 2.0, and 2.5 mg mL^−1^ solutions. The semi-refined polysaccharide was dissolved in distilled water to prepare 0.6, 1.2, 1.8, 2.4, and 3.0 mg mL^−1^ solutions. The ABTS scavenging activity was estimated as described by Li et al. (2012) [26]. Briefly, a mixed solution of 7.4 mM ABTS diammonium salt and 2.6 mM potassium persulfate (1:1, *v*/*v*) was prepared and maintained in the dark for 12 h. This was then diluted with phosphate buffer (pH 7.4) to A_734_ nm of 0.70. The diluted ABTS solution (0.8 mL) was mixed with samples of different concentrations with mild shaking in the dark at 37 °C for 15 min. The A_734_ of the reaction mixture was measured by an Epoch™ 2 Microplate Spectrophotometer (Bio-Tek Instruments Inc., Winooski, VT, USA). The ABTS radical scavenging ability was calculated as follows:

Scavenging activity of ABTS (%) = (A_0_ − A_1_)/A_0_ × 100% (5)

where A_0_ and A_1_ are the absorbance of the control and treatment samples, respectively.

#### 2.8.2. DPPH Radical Scavenging Activity

The crude phycoerythrin was dissolved in distilled water to prepare 0.4, 0.8, 1.2, 1.6, and 2.0 mg mL^−1^ solutions. The crude lipid was dissolved in ethanol to prepare 0.1, 0.2, 0.3, 0.4, and 0.5 mg mL^−1^ solutions. The semi-refined polysaccharide was dissolved in distilled water to prepare 0.2, 0.4, 0.6, 0.8, and 1.0 mg mL^−1^ solutions. The corresponding samples of different concentrations were added to 0.1 μM DPPH ethanol solution in equal volume. The reaction was performed at RT for 30 min in the dark. The absorbance was determined at 517 nm using an Epoch™ 2 Microplate Spectrophotometer (Bio-Tek Instruments Inc., Winooski, USA). The DPPH scavenging activity of the mixture was determined by the following formula [27]:

Scavenging activity of DPPH (%) = (1 − (A_1_ − A_2_)/A_0_) × 100% (6)

where A_0_, A_1_, and A_2_ are the absorbance of the control (sample solution prepared with distilled water), experimental (sample solution), and blank (ethanol instead with DPPH) groups, respectively.

#### 2.8.3. Superoxide Anion Radical Scavenging Activity

The crude phycoerythrin was dissolved in distilled water to prepare 0.4, 0.8, 1.2, 1.6, and 2.0 mg mL^−1^ solutions. The crude lipid was dissolved in ethanol to prepare 0.2, 0.4, 0.6, 0.8, and 1.0 mg mL^−1^ solutions. The semi-refined polysaccharide was dissolved in distilled water to prepare 1.0, 2.0, 3.0, 4.0, and 5.0 mg mL^−1^ solutions. The superoxide anion scavenging activity was measured using the commercially available test kit (Nanjing Jiancheng Bioengineering Research Institute, Nanjing, China). The superoxide anion scavenging activity of the extract was calculated as follows:

Scavenging activity of superoxide anion (%) = (1 − (A_1_/A_0_)) × 100% (7)

where A_0_ and A_1_ are the absorbance of the control (prepared with distilled water) and the treatment samples, respectively.

### 2.9. Fourier-Transform Infrared Spectroscopy (FTIR)

IR Affinity-1 Fourier-transform infrared spectrometer (Shimadzu, Kyoto, Japan) was employed to determine the polysaccharide with the scanning interval of 400–4000 cm^−1.^

### 2.10. Spectral Characteristics of Crude Phycoerythrin

The absorbance (200–800 nm) of the crude phycoerythrin extracts was measured using a TU-1810 spectrophotometer (Beijing Persee General Instrument Co. Ltd., Beijing, China). The two-dimensional fluorescence spectrum at 200–800 nm was observed by an F97 PRO fluorescence spectrophotometer (Lengguang Technology, Shanghai, China).

### 2.11. Statistical Analysis

All treatments had three independent biological repeats and each measurement was performed in triplicate. One-way analysis of variance (ANOVA) was performed to determine the significant differences between the target datasets using SPSS version 18.0 software (SPSS, Chicago, IL, USA).

## 3. Results

### 3.1. Biomass Reduction during Three-Step Extraction

**Figure 1:**
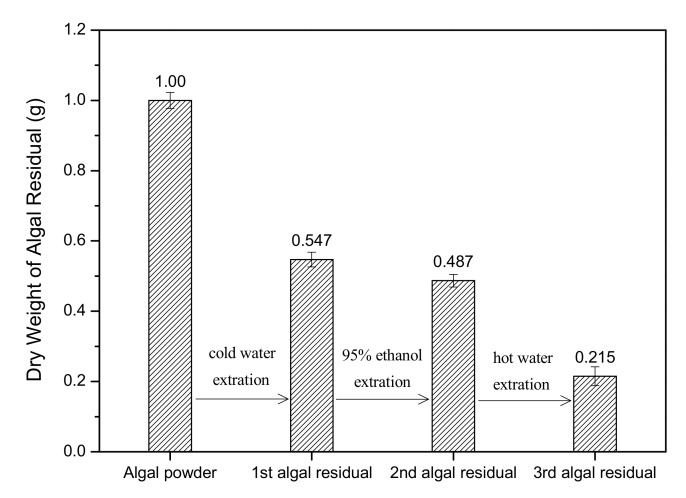
Biomass reduction during three-step extraction process (*n* = 3; data: mean ± SD).

After phycoerythrin extraction using cold water (4 °C), 1.0 g of *Porphyridium* biomass produced 0.547 g of the 1st algal residue (freeze-dried), suggesting a reduction of 45.3% (0.453 g) in the biomass DW (Figure 1). Subsequently, the 1st algal residue was extracted with 95% ethanol at RT for 6 h, which produced 0.487 g of the 2nd algal residue with a further loss of 6.0% (0.06 g) in DW. Finally, the 2nd algal residue was extracted with deionized water at 80 °C for 3 h, which produced 0.215 g of the 3rd algal residue, with a further loss of 27.2% (0.272 g) in DW. After the three-step extraction, the total biomass was reduced by 78.5% in DW.

### 3.2. Biochemical Composition of the Reduced Biomass during Three-Step Extraction

**Figure 2:**
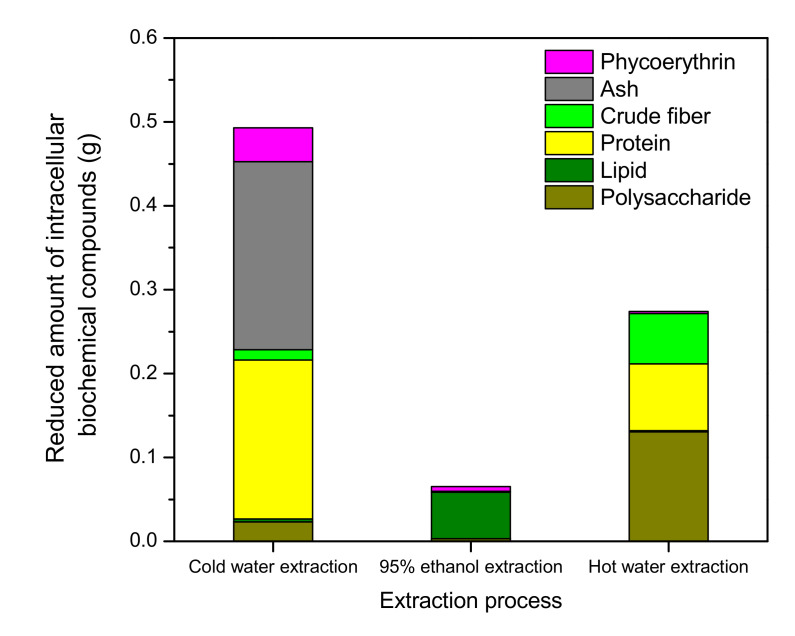
Biochemical composition of the reduced biomass during the three-step extraction process (*n* = 3; data: mean ± SD).

Later, the biochemical components of the reduced biomass were determined at every step of the three-step extraction process (Figure 2). After the first step (cold water extraction), the content of polysaccharides, lipids, proteins, crude fibers, ash, and phycoerythrin in the biomass decreased by 2.3, 0.3, 19.0, 1.2, 22.4, and 4.1%, respectively. Ash and proteins were the two most-reduced substances, followed by phycoerythrin. After the second step (95% ethanol extraction), the biomass contents of polysaccharides, lipids, proteins, crude fibers, ash, and phycoerythrin decreased by 0.3, 5.5, 0.1, 0, 0, and 0.6%, respectively. Lipid was the most reduced substance in the second step. After the final extraction step (hot water extraction), the respective contents of polysaccharides, lipids, proteins, crude fibers, ash, and phycoerythrin in the biomass decreased by 13.0, 0.2, 8.0, 6.0, 0, and 0.2%. Overall, polysaccharides were the most decreased substances, followed by proteins and crude fibers after the final step.

The contents of intracellular phycoerythrin, polysaccharides, and total lipids in the original biomass were 4.9, 7.0, and 17.8%, respectively. After the three-step extraction process, the obtained amounts of crude phycoerythrin, crude lipid, and intracellular crude polysaccharides were 0.031 (yield: 63.3%), 0.052 (yield: 74.3%), and 0.134 g (yield: 75.2%), respectively (Table 1).

### 3.3. Spectral Characteristics of Crude Phycoerythrin

**Figure 3:**
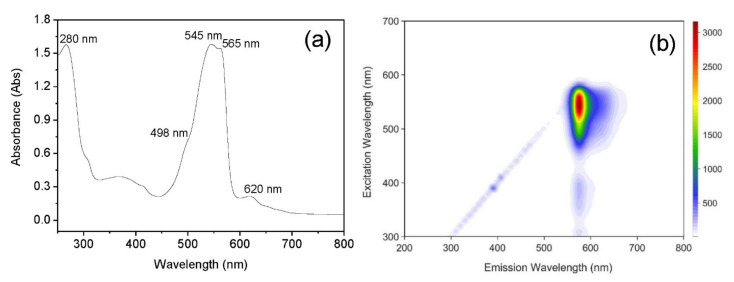
Spectral characteristics of the crude extract of *Porphyridium purpureum*. (**a**) UV–Vis absorption spectrum (200–800 nm) and (**b**) two-dimensional fluorescence spectrum (200–900 nm).

The absorption spectrum (250–800 nm) of crude phycoerythrin extract showed two major peaks at 545 and 565 nm and a shoulder peak at 498 nm (Figure 3a). All of these corresponded to B-phycoerythrin. However, a small absorption peak at 620 nm was observed for phycocyanin and a peak at 280 nm was observed for proteins. The purity of crude phycoerythrin extract calculated based on the A_545_/A_280_ ratio was 1.201. This suggests that the low purity of the crude extract needs attention during downstream processing even though cold water can extract phycoerythrin.

The two-dimensional fluorescence spectrum (Figure 3b) of the crude phycoerythrin extract showed the highest intensity emission peak at 576 nm (excitation 545 nm), which was consistent with the fluorescence characteristics of B-phycoerythrin.

### 3.4. Lipomics Analysis of Ethanol Extract

**Figure 4:**
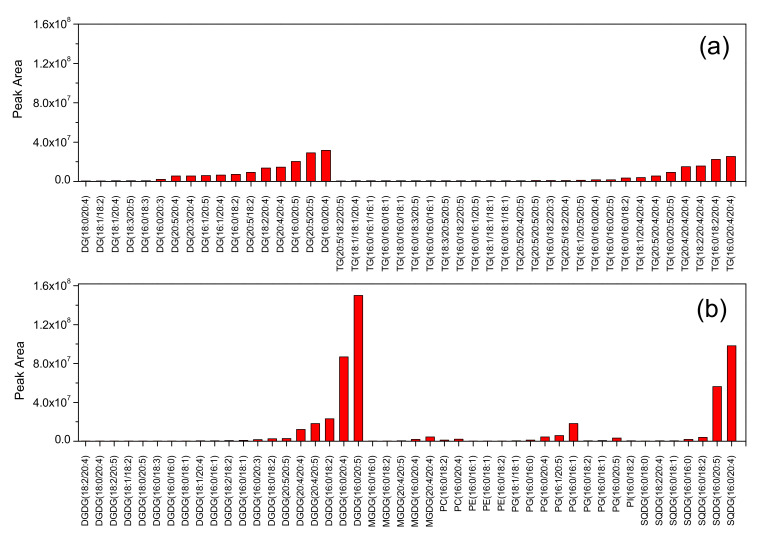
Lipomics analysis of crude lipid of *Porphyridium purpureum*. (**a**) neutral lipid and (**b**) polar lipid. digalactosyldiacylglycerol (DGDG), monogalactosyldiacylglycerol (MGDG), sulphoquinovosyldiacylglycerol (SQDG), phosphatidylglycerol (PG), diglyceride (DG), triglyceride (TG), and phosphatidylethanolamine (PE).

Membrane and storage lipids are the primary lipids of *Porphyridium*. As depicted in Figure 4, the top 10 high proportion membrane lipids were digalactosyldiacylglycerol (DGDG) (C16:0/C20:5), sulphoquinovosyldiacylglycerol (SQDG) (C16:0/C20:4), DGDG (C16:0/C20:4), SQDG (C16:0/C20:5), DGDG (C16:0/C18:2), DGDG (C20:4/C20:5), phosphatidylglycerol (PG) (C16:0/C16:1), DGDG (C20:4/C20:4), PG (C16:1/C20:5), and monogalactosyldiacylglycerol (MGDG) (C20:4/C20:4). Similarly, the top 10 high proportion storage lipids were diacylglycerol (DAG) (C16:0/20:4), DAG (C20:5/20:5), triacylglycerol (TAG) (C16:0/20:4/20:4), TAG (C16:0/C18:2/C20:4), DAG (C16:0/C20:5), TAG (C18:2/C20:4/C20:4), TAG (C20:4/C20:4/C20:4), DAG (C20:4/C20:4), DAG (C18:2/C20:4), and TAG (C16:0/C20:5/C20:5). The peak areas of different lipid molecules corresponding to DGDG and SQDG were C20:4 and C20:5 fatty acids. This indicates a strong distribution of C20:4 and C20:5 fatty acids in the *Porphyridium* membrane.

### 3.5. Characteristics of Polysaccharides in Porphyridium

**Figure 5:**
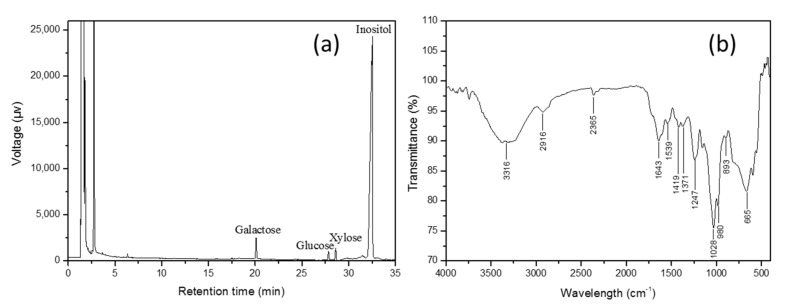
Characteristics of semi-refined polysaccharides in *Porphyridium purpureum*. (**a**) Monosaccharide composition and (**b**) Fourier infrared spectra.

Galactose (39.58%), xylose (38.83%), and glucose (21.59%) were the major monosaccharide components of the polysaccharides (Table 2 and Figure 5a). The unique absorption peaks of saccharide at 3200–3600 cm^−1^ (3316 cm^−1^) representing the O-H stretching vibration, and 2800–3000 cm^−1^ (2916 cm^−1^) indicating the C-H stretching vibration were evident (Figure 5b). A strong absorption peak near 1730–1630 cm^−1^ corresponding to the C=O stretching vibration of -CHO was also observed. The characteristic peak of -COOH stretching at 1419 cm^−1^ indicated the presence of glucuronic acid. However, the absence of an absorption peak at 1775–1735 cm^−1^ suggested the absence of acetyl polysaccharides in *Porphyridium*. The C–-O stretching vibration peak at 1000–1200 cm^−1^ (1028 cm^−1^), and an absorption peak at 893 cm^−1^ indicated the presence of alpha and beta glycoside bonds in the *Porphyridium* polysaccharides.

The biochemical components of the polysaccharides showed 55.57% purity. Though the polysaccharides were deproteinized several times, some proteins (8.01% DW) remained covalently bound. The contents of glucuronic acids and sulfate radicals were 4.13 and 8.31% DW, respectively (Table 3). The highest sulfate radical content in the intracellular polysaccharides indicated potential biological activities.

### 3.6. Antioxidant Activity of the Respective Extracts of Porphyridium

**Figure 6:**
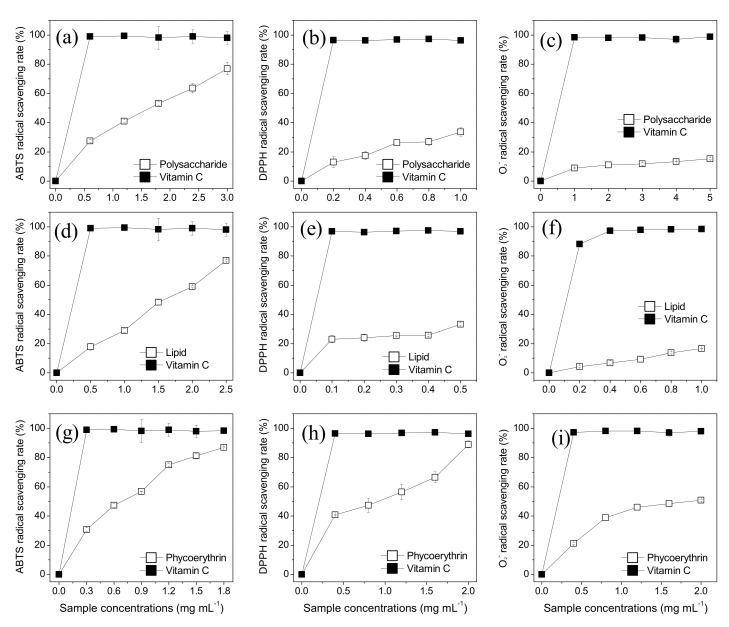
Antioxidant activities of the three extracts of *Porphyridium purpureum*. (**a**) ABTS scavenging activity of polysaccharide, (**b**) DPPH scavenging activity of polysaccharide, (**c**) superoxide anion scavenging activity of polysaccharide, (**d**) ABTS scavenging activity of crude lipids, (**e**) DPPH scavenging activity of crude lipids, (**f**) superoxide anion scavenging activity of crude lipids, (**g**) ABTS scavenging activity of crude phycoerythrin, (**h**) DPPH scavenging activity of crude phycoerythrin, and (**i**) superoxide anion scavenging activity of crude phycoerythrin (*n* = 3; data: mean ± SD).

The crude phycoerythrin, crude lipid, and semi-refined polysaccharide showed concentration-dependent ABTS scavenging activity (Figure 6a,d,g) with the corresponding IC_50_ of 0.53, 1.53, and 1.43 mg mL^−1^, respectively (Table 4). Notably, the antioxidant activity of crude phycoerythrin was the highest. Similarly, the crude phycoerythrin showed the strongest DPPH scavenging activity compared to crude lipid and semi-refined polysaccharides (Figure 6b,e,h). Phycoerythrin at 2.0 mg mL^−1^ showed the highest DPPH scavenging rate of 89.0%. IC50 of the crude phycoerythrin, crude lipid, and semi-refined polysaccharide were 0.69, 0.87, and 1.90 mg mL^−1^, respectively (Table 4). The semi-refined polysaccharide and crude lipid at 5.0 and 1.0 mg mL^−1^ showed a scavenging rate of only 20%. The IC_50_ of crude phycoerythrin, crude lipid, and semi-refined polysaccharide of superoxide anion were 1.56, 5.18, and 23.47 mg mL^−1^, respectively (Table 4). Phycoerythrin again showed the highest scavenging activity.

## 4. Discussion

Microalgae can accumulate a variety of bioactive components. Therefore, increasing the utilization efficiency, such as zero waste multiproduct biorefinery for *Arthrospira* sp., has become the research hotspot [28]. In recent years, *P. purpureum* has emerged as a widely accepted biomass due to its high-value components (B-phycoerythrin, PUFAs, and polysaccharides). Gallego et al. (2019) extracted phycoerythrin, polysaccharides, and carotene from *Porphyridium* using a multi-step compressed fluids-based technique; however, the process required operation at 10.5 MPa [29]. In contrast, our study developed an extraction process that can be performed at atmospheric pressure. Herein, a sequential extraction process based on the characteristics of phycoerythrin, PUFAs, and polysaccharide, was established using cold water, 95% ethanol, and hot water.

In our method, the extraction yield of phycoerythrin was low at 64.0%. However, previous studies suggest that an increase in the extraction time or some auxiliary extraction methods could improve the extraction yield of phycoerythrin. Martinez et al. (2019) employed pulsed electric field treatments (PEF, 8 or10 kV cm^−1^ for 150 μs) to improve the extraction efficiency of phycoerythrin [30]. Tran et al. (2019) improved the extraction rate using high hydrostatic pressure. However, a pressure of 400 MPa affected the fluorescence characteristic of phycoerythrin [31]. In our study, the presence of other proteins reduced the purity of the crude phycoerythrin extract (A_545_/A_280_ = 1.201). Tang et al. (2016) improved phycoerythrin purity from 0.9 to 5.1 using a SOURCE 15Q anion exchange column, with a yield of 68.5% [32]. This suggests that a downstream auxiliary extraction method could improve the purity and yield of phycoerythrin. High-purity phycoerythrin has been widely adopted as a fluorescent dye for flow cytometry due to its excellent fluorescence characteristics, but no low-purity phycoerythrin is available on the market [4]. Large red algae are the primary source of phycoerythrin. However, a large amount of carrageenan in the red algae severely affects the separation and purification of phycoerythrin, resulting in a high cost of phycoerythrin. At present, phycocyanin from *Spirulina* has been widely accepted in the market as pigments and health products [3]. The price of food-grade phycocyanin with a color value of E25 is USD 201.0/kg [33]. The strong coloring, anti-oxidation, and anti-cancer abilities of phycoerythrin are similar to phycocyanin. The estimated market value of phycoerythrin is also very large. In the future, low-purity phycoerythrin might be targeted in the following major markets: (1) natural pigments (natural water-soluble pink pigments are relatively scarce and emerging health concerns demand the replacing of synthetic pink pigments) and (2) health care products (phycoerythrin could be a potential health product due to its excellent antioxidant and anticancer abilities).

The crude lipid of *Porphyridium* appeared black in color and had poor fluidity. Some vegetable oils (e.g., olive oil) possess excellent fluidity, while the crude lipid of *Porphyridium* has no fluidity at all. Similar to butter, the crude lipid never flows out even if the container is turned upside down. The poor fluidity might be attributed to a large portion of membrane lipids, remaining solid at RT [17]. The black color might be attributed due to the color of chlorophyll (or chlorophyllin, the degraded product) and β-carotene as the green chlorophyll and orange β-carotene were mixed together to produce a black effect [17]. Chlorophyll and β- carotene are fat-soluble pigments and difficult to separate from triacylglycerol [24]. Therefore, the crude lipid extract needed downstream purification to meet the requirements of commercial application. DGDG (C16:0/C20:5), DGDG (C16:0/C20:4), SQDG (C16:0/C20:4), and SQDG (C16:0/C20:5), the components of the chloroplast membrane, account for a high proportion of *Porphyridium* lipids (Figure 4). Recent studies have reported that glycolipid-containing PUFAs have excellent biological activity. Yang et al. (2019) reported that ω-6 PUFAs containing glycolipids from *Spirulina* alleviated skin injury in Zebrafish [34]. Notably, the glycolipids of *Porphyridium* contain EPA and ARA. However, their biological activities have not yet been reported. Additionally, EPA in *Porphyridium* was found to be mostly distributed in DGDG and SQDG, which was consistent with the previous studies. Khozin-Goldberg et al. (2000) showed that EPA accounts for 47% of the total fatty acids in *P. cruentum*, but only 2% EPA was distributed in triacylglycerol [35]. EPA is largely distributed in the membrane lipids in most microalgae species, such as *Nannochloropsis oceanica* [36] and *Phaeodactylum tricornutum* [37]. A high EPA distribution into membrane lipids increases membrane fluidity, making algal adaptation easy to the environment [38].

Microalgal polysaccharides can be extracted with acid and alkali solutions. However, these can damage the inherent structure of polysaccharides [16]. Therefore, in this study, we used water extraction to maintain the structural stability of polysaccharides. The polysaccharide of *Porphyridium* was yellow in color with poor water solubility. Consistent with an extracellular polysaccharide, galactose (39.58%), xylose (38.83%), and glucose (21.59%) were found to be the major monosaccharide components, which differed in content (galactose 21.0%, xylose 36.0%, and glucose 23.0%) from the reports of Bernaerts et al. (2018) [39]. The synthesis of intracellular and extracellular polysaccharides can be linked as the repeat units of polysaccharides are determined by glycosyltransferases [40]. Also, the intracellular polysaccharides contained 8.31% of sulfate radicals, explaining their antioxidant activity [41]. Notably, the intracellular polysaccharides maintained 8.01% proteins even after deproteinization (Table 3), suggesting covalent interactions between them. A previous study showed that extracellular polysaccharides contained a 66 kDa protein with high mannose (8–9 residues) content and participated in the polysaccharide synthesis [42]. However, it is uncertain whether the covalently bound protein(s) in the current study have a similar structure and could participate in intracellular polysaccharide synthesis.

Furthermore, the antioxidant activities of the three extracts varied to a great extent. Crude phycoerythrin showed the highest antioxidant activity, followed by crude lipid and intracellular polysaccharides. The crude extract is a complex system consisting of several other compounds. For instance, there could be various water-soluble small-molecular compounds, such as floridosides, in the crude phycoerythrin extract [43]. In addition to glycerides, the crude lipid extract has many fat-soluble compounds, such as chlorophyll [17], which can affect antioxidant activity. High molecular weight intracellular polysaccharides with complex structures could encapsulate certain active groups, thereby weakening the antioxidant activity [44]. Some auxiliary treatments, such as hermetical microwave treatment, could significantly increase the antioxidant activity of polysaccharides [44]. However, it is necessary to purify the components to the highest purity, instead of comparing the crude extracts obtained in this study for a better comparison of antioxidant activities, as the prime focus of this study was to establish the extraction method.

*Porphyridium* has not been officially accepted by the US FDA as “generally recognized as safe” (GRAS). However, accumulating studies have reported the feasibility of *Porphyridium* as an animal feed/human food application. Safi et al. (2013) reported that the amino acid sequence of the biomass and the protein extract of *Porphyridium* meet the standard protein/amino acid requirements proposed by the Food and Agricultural Organization and World Health Organization [45]. Kavitha et al. (2016) assessed the acute and subchronic safety of *Porphyridium* biomass in albino Wistar rats and its application as animal feed/human food. The results showed that the biomass smentation did not induce any abnormality in body weight gain, relative organ weights, histopathology, or hematological and serum biochemical indices, instead the biomass was well tolerated in the rats assuring its nontoxicity [46]. Additionally, many studies have reported the active components of *Porphyridium* as anti-tumor drugs [1]. Therefore, it was proposed that *Porphyridium* could become a potential human superfood smentation and the main feedstock for phycoerythrin production in the future. Similar fractional extraction strategies could be developed for industrialized algae strains, such as *Chlorella, Spirulina*, *Dunaliella salina*, and *Haematococcus pluvislis*, to achieve zero waste with increased utilization efficiency.

## 5. Conclusions

In conclusion, a feasible three-step fractional extraction process was established for phycoerythrin, lipid, and polysaccharide extraction from *Porphyridium* biomass using cold water, 95% ethanol, and hot water. The total extraction rate was 78.5%, with reduced purity. The crude phycoerythrin showed excellent fluorescence characteristics. The crude lipid contained a large number of membrane lipids, including DGDG and SQDG. The intracellular polysaccharides were mainly composed of glucose, xylose, and galactose. Among all three major components, crude phycoerythrin showed the highest antioxidant activity.

## Figures and Tables

**Table 1:** The yield of phycoerythrin, lipid, and polysaccharide in the fractional extraction.

	Algal Powder	Phycoerythrin	Lipid	Polysaccharide
Content (% DW)	/	4.9 ± 0.1	7.0 ±0.2	17.8 ± 0.05
Theoretical weight (g)	1.000	0.049 ± 0.001	0.070 ± 0.002	0.178 ± 0.001
Actual weight (g)	1.000 ± 0.002	0.031 ± 0.001	0.052 ± 0.003	0.134 ± 0.016
Yield (%)	/	63.3 ± 2.7	74.3 ± 4.0	75.2 ± 8.8

DW: dry weight.

**Table 2:** Monosaccharide composition of intracellular polysaccharides.

	Xylose	Glucose	Galactose
Percentage (%total carbohydrate)	38.83 ± 0.56	21.59 ± 0.79	39.58 ± 1.23

**Table 3:** Analysis of biochemical composition of intracellular polysaccharides.

	Carbohydrate	Protein	Uronic Acid	SO_4_^2−^
Content (%DW)	55.57 ± 0.85	8.01 ± 0.02	4.13 ± 0.05	8.31 ± 0.19

**Table 4:** IC_50_ of three crude extracts.

IC_50_ (mg mL^−1^)	Phycoerythrin	Lipid	Polysaccharide	Vitamin C (Control)
ABTS	0.53 ± 0.05 ^b1^	1.53 ± 0.09 ^a1^	1.43 ± 0.02 ^a1^	0.01 ± 0.00 ^c1^
DPPH	0.69 ± 0.02 ^c2^	0.87 ± 0.06 ^b2^	1.90 ± 0.09 ^a2^	0.01 ± 0.00 ^d2^
Superoxide anion	1.56 ± 0.01 ^c3^	5.18 ± 0.16 ^b3^	23.47 ± 1.65 ^a3^	0.02 ± 0.00 ^d3^

ABTS: 2, 2′-azinobis (3-ethylbenzothiazoline-6-sulfonic acid; DPPH: 2, 2-Diphenyl-1-picrylhydrazyl; IC_50_: half maximal inhibitory concentration. Different letters denoted significant differences among the IC_50_ values of the phycoerythrin, lipid, polysaccharide, and vitamin C (^a1–c1^: ABTS;^ a2–d2^: DPPH; ^a3–d3^: Superoxide anion).

## Data Availability

The study did not report any data.
